# Association between LDL-C/HDL-C ratio and first stroke in hypertensive patients: a prospective cohort study

**DOI:** 10.3389/fendo.2025.1657213

**Published:** 2025-09-11

**Authors:** Chao Yu, Meihui Wu, Lingjuan Zhu, Tao Wang, Weifang Zhang, Wei Zhou, Huihui Bao, Xiaoshu Cheng

**Affiliations:** ^1^ Center for Prevention and Treatment of Cardiovascular Diseases, The Second Affiliated Hospital of Nanchang University, Nanchang, Jiangxi, China; ^2^ Jiangxi Provincial Cardiovascular Disease Clinical Medical Research Center, Nanchang, Jiangxi, China; ^3^ Department of Pharmacy, The Second Affiliated Hospital of Nanchang University, Nanchang, Jiangxi, China; ^4^ School of Pharmacy, Jiangxi Science and Technology Normal University, Nanchang, Jiangxi, China; ^5^ Department of Cardiovascular Medicine, The Second Affiliated Hospital of Nanchang University, Nanchang, Jiangxi, China

**Keywords:** hypertension, LDL-C/HDL-C ratio, first stroke, cohort study, alcohol drinking

## Abstract

**Background:**

Existing evidences regarding the association between the ratio of low-density lipoprotein cholesterol (LDL-C) to high-density lipoprotein cholesterol (HDL-C) and first stroke in hypertensive patients remains limited. This study aims to assess the role of LDL-C/HDL-C ratio in the risk of first stroke in Chinese hypertensive patients.

**Methods and results:**

This prospective cohort study encompassed 12, 893 hypertensive patients from the Chinese Hypertension Registry. Cox proportional hazards regression, restricted cubic spline (RCS), and subgroup analysis were applied to evaluate the association between LDL-C/HDL-C ratio and first stroke. The hazard ratio (HR) and 95% confidence interval (CI) were used to estimate the strength of the association. The mean age of all participants was 63.7 ± 9.5 years, and 531 cases of first stroke occurred, with an average follow-up time of 3.9 years. In the fully adjusted model, each 1-unit increase of LDL-C/HDL-C ratio raised the risk of first stroke by 43% (HR=1.43, 95% CI: 1.22–1.67). Compared with patients in the Q1 of LDL-C/HDL-C ratio, the adjusted HRs of stroke for those in Q2, Q3, and Q4 were respectively 1.32 (95% CI: 1.03,1.70), 1.49 (95% CI: 1.14, 1.96), and 1.94 (95% CI: 1.45, 2.59), with a statistically significant trend (P for trend < 0.001). Analyses using restricted cubic spline confirmed the linear association between the LDL-C/HDL-C ratio and first stroke. Subgroup analysis revealed a stronger association between the LDL-C/HDL-C ratio and first stroke in drinkers (P for interaction=0.024).

**Conclusion:**

A high LDL-C/HDL-C ratio may increase the risk of first stroke in hypertensive patients, especially among current drinkers.

## Introduction

Stroke is the leading cause of disability and death worldwide (1). Between 1990 and 2019 (2), global stroke prevalence cases, incidence cases, and deaths increased significantly by 85%, 70%, and 43%, respectively. The incidence and prevalence of stroke in China continue to rise (3–6), making it a major public health challenge. According to the latest epidemiologic survey data (7), China has 2.4 million new stroke cases and 1.1 million stroke-related deaths each year. Globally, hypertension is not only one of the most important risk factors for stroke (8, 9) but also a primary risk factor for ischemic stroke and hemorrhagic stroke (10). Given that traditional risk factors do not fully explain stroke risk, identifying and intervening with modifiable risk factors, especially in hypertensive patients, is essential to optimize stroke prevention and reduce the burden of disease and the risk of recurrence.

Growing evidence suggests that the ratio of low-density lipoprotein cholesterol (LDL-C) to high-density lipoprotein cholesterol (HDL-C) is a new indicator of atherosclerotic cardiovascular disease risk (11, 12). This ratio not only reflects the state of lipoprotein homeostasis (13), but also is significantly associated with the risk of cardiovascular events (14, 15). Specifically, elevated LDL-C levels are a clear risk factor for ischemic stroke and further increase the risk of subsequent major cardiovascular events, especially in patients with a history of stroke (16, 17). Low HDL-C levels, on the other hand, independently affect the prognosis of cerebrovascular disease (18–20), and studies have confirmed that HDL-C is negatively associated with the risk or severity of ischemic stroke (21, 22).

Nevertheless, existing evidence regarding the association between LDL-C/HDL-C ratio and stroke risk in hypertensive populations remains limited, and stroke etiology involves multifactorial mechanisms. This study was therefore conducted to examine the relationship between LDL-C/HDL-C ratio and stroke in Chinese hypertensive patients, aiming to provide evidence for stroke prevention strategies, therapeutic approaches, and comprehensive clinical management.

## Methods

### Participants

All data used in this study were obtained from the Chinese Hypertension Registry Study (registration number: ChiCTR1800017274), which was a real-world, multicenter, observational study. The study was initiated in March 2018 in Wuyuan County, Jiangxi Province, China, and the inclusion criteria were (1) aged ≥18 years and (2) having hypertension, defined as sitting resting blood pressure ≥140/90 mm Hg or taking antihypertensive drugs. The study aimed to assess the current treatment status, associated risk factors, and prognosis of local hypertensive patients. Specific inclusion and exclusion criteria have been detailed previously (23). This study was conducted in accordance with the Declaration of Helsinki and approved by the Ethics Committee of the Institute of Biomedical Sciences, Anhui Medical University (NO. CH1059), and the Second Affiliated Hospital of Nanchang University (NO. 2018019). All patients signed an informed consent before enrollment.

**Figure 1:**
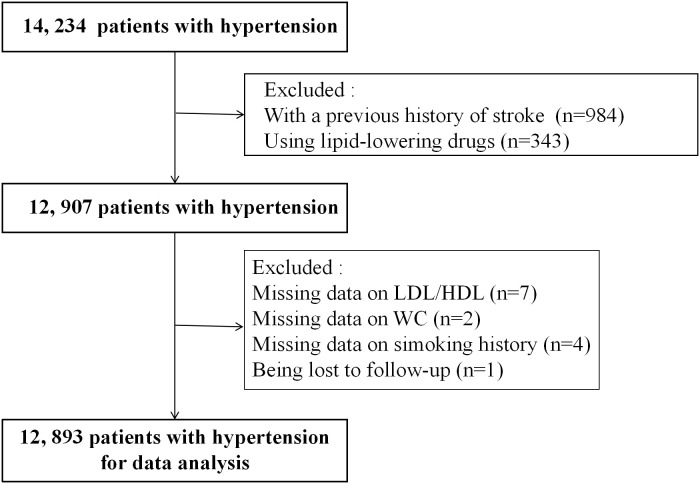
Flow chart of study participants.

A total of 14,234 hypertensive patients were enrolled in this study. Patients with a previous history of stroke (n = 984), using lipid-lowering drugs (n = 343), missing data on HDL-C/LDL-C (n = 7), missing data on waist circumference (WC) (n = 2), missing data on smoking history (n = 4), and being lost to follow-up (n = 1) were excluded. Finally, 12,893 subjects were analyzed (**Figure 1**).

### Data collection

All participants underwent baseline and follow-up health assessments by trained researchers following standard operating procedures to determine their demographic characteristics, including age, sex, smoking history, alcohol consumption, medical history, and medications. Anthropometric indicators include height, weight, and waist circumference. Body mass index (BMI) is defined as weight in kilograms divided by height in meters squared (kg/m²). Trained medical personnel assessed blood pressure (BP) to control for measurement differences between different observers. After the participants were allowed to rest for 5 min, systolic blood pressure (SBP), diastolic blood pressure (DBP), and heart rate (HR) were measured three times consecutively with an Omron electronic sphygmomanometer. The interval between consecutive readings was 1 minute, and three measurements were taken on the right arm and averaged. With respect to alcohol drinking, subjects were classified as never, former or current drinkers, and according to alcohol consumption (none, ≤2 drinks per week, ≥2 drinks per week) (24). Smoking status was defined as never (smoked <100 cigarettes in lifetime), former (smoked ≥100 cigarettes in lifetime but had not reported smoking at baseline), or current smoking (smoked cigarettes in the past 30 days) (25).

Subjects were informed in advance of the need to fast overnight for 8–12 hours before blood samples were collected. The samples were quickly processed to obtain serum, which was stored at -80 °C for testing (Bioengineering, Shenzhen, Guangdong, China). All tests were performed using a fully automated clinical analyzer (Beckman Coulter, USA) to measure fasting blood glucose (FPG), homocysteine (Hcy), triglycerides (TG), total cholesterol (TC), HDL-C and LDL-C. The estimate glomerular filtration rate (eGFR) was calculated using the Chronic Kidney Disease Epidemiology Collaboration (CKD-EPI) equation (26). Chronic kidney disease (CKD) was defined as eGFR < 60 mL/min/1.73 m^2^ (27). Dyslipidemia was defined as TC greater than or equal to 5.2mmol/L, TG greater than or equal to 1.7 mmol/L, LDL-C greater than or equal to 3.4 mmol/L,or HDL-C less than 1.0 mmol/L, or self-reported dyslipidemia (28).

Diabetes mellitus was defined as a self-reported physician diagnosis of diabetes mellitus, FPG concentration ≥ 7.0 mmol/L, or use of hypoglycemic medications. The history of coronary heart disease (CHD) was primarily self-reported by participants through a questionnaire. Each participant was asked about the presence of symptoms, the type of treatment received, and the availability of medical records (including discharge summaries, biochemical test data, and imaging data) during episodes of coronary heart disease or stroke atrial fibrillation (AF) was defined as the presence of AF on a standard 12-lead electrocardiogram or a history of AF.

### Outcome assessments

The primary outcome of follow-up was the first occurrence of a nonfatal or fatal symptomatic stroke, excluding subarachnoid hemorrhage and subclinical stroke. Outcome information was initially collected through face-to-face questionnaire surveys, home visits, or telephone interviews, and subsequently verified using hospital information systems (HIS), the National Health Information Platform, or the National Basic Medical Insurance System to ensure complete ascertainment of stroke events across the entire cohort. Final adjudication of all suspected stroke cases was conducted by the End Point Adjudication Committee, which comprised experts in neurology, neurosurgery, cardiology, cardiovascular surgery, and public health, applying predefined diagnostic criteria.

### Statistical analyses

Baseline characteristics were described using mean (standard deviation, SD) for continuous variables. Categorical variables were described as frequency (%). One-way analysis of variance or chi-square tests were used to compare the characteristics of populations grouped by LDL-C/HDL-C ratio quartiles and to explore the distribution of the intervals.

Cox proportional hazard regression was used to estimate hazard ratio (HR) and 95% confidence interval (CI) for the association between the LDL-C/HDL-C ratio and the first stroke. Three models were constructed with sequential adjustments: Model 1 was adjusted no covariates. Model 2 was adjusted for sex and age. Model 3 was adjusted for sex, age, BMI, WC, SBP, DBP, FPG, TC, TG, eGFR, Hcy, smoking, drinking, diabetes, dyslipidemia, CKD, CHD, AF, antiplatelet drugs, glucose-lowering drugs. In the regression analyses model, covariates were selected based on clinical importance, statistical significance in univariable analysis, and the potential confounders effect estimates individually changed by at least 10%. Multiple collinearity screening between covariates showed that all variance inflation factors (VIF) were less than 5. The proportional hazards assumption was verified using Schoenfeld residual tests. A generalized additive model with restricted cubic spline smoothing was employed to evaluate the dose-response relationship between the LDL-C/HDL-C ratio and first stroke. Subgroup analyses were performed to assess potential effect modifications in the association between the LDL-C/HDL-C ratio and stroke. To control the overall type I error, the Bonferroni method was applied for multiple testing correction: the adjusted significance level α for the interaction tests in stratified subgroups was set at 0.05/7 = 0.007.

All statistical analyses were performed using R software (version 3.3.1 version; http://www.R-project.org) and Empower version 2.17.8 (www.empowerstats.com) for statistical analysis. All statistical tests were performed using a two-sided test and were statistically significant with a P value < 0.05.

## Results

### Baseline characteristics of study participants

A total of 12, 893 hypertensive patients (mean age 63.7 ± 9.5 years; 46.5% males) were finally enrolled for the final analysis. Baseline characteristics of the patients grouped by LDL-C/HDL-C quartiles were presented in **Table 1**. Participants with higher LDL-C/HDL-C ratio were more likely to be younger, females, have higher BMI, WC, DBP, FPG, Hcy, TC, TG, higher rates of current smoking, diabetes, use of antihypertensive drugs and glucose-lowering drugs, have lower eGFR, and lower rates of current drinking and AF. SBP, CKD, CHD, use of antiplatelet drugs did not differ statistically between LDL-C/HDL-C quartiles.

**Table 1:** Baseline characteristics of the study population according to LDL-C/HDL-C ratio quartiles.

Characteristics	Total	LDL-C/HDL-C				P-value
		Q1 0.21-1.54	Q2 1.55-1.96	Q3 1.97-2.42	Q4 2.43-6.51	
N	12893	3203	3223	3247	3220	
Age, years	63.7 ± 9.5	66.0 ± 9.2	64.0 ± 9.2	63.0 ± 9.3	61.8 ± 9.6	<0.001
Male, n (%)	5999 (46.5)	1709 (53.4)	1432 (44.4)	1332 (41.0)	1526 (47.4)	<0.001
BMI, kg/m^2^	63.7 ± 9.5	21.7 ± 3.4	23.3 ± 3.3	24.3 ± 3.3	25.0 ± 3.3	<0.001
WC, cm	83.8 ± 9.9	78.3 ± 9.6	82.9 ± 9.1	85.8 ± 9.1	88.1 ± 8.8	<0.001
SBP, mmHg	148.7 ± 17.7	149.0 ± 18.2	149.0 ± 17.6	148.7 ± 17.5	148.2 ± 17.6	0.225
DBP, mmHg	89.2 ± 10.7	87.8 ± 11.0	89.0 ± 10.7	89.3 ± 10.6	90.5 ± 10.6	<0.001
FPG, mmol/L	6.2 ± 1.6	5.9 ± 1.2	6.1 ± 1.5	6.2 ± 1.5	6.5 ± 2.0	<0.001
Hcy, μmol/L	17.8 ± 10.8	17.9 ± 10.2	17.5 ± 10.6	17.5 ± 10.5	18.3 ± 11.9	0.004
TC, mmol/L	5.2 ± 1.1	4.8 ± 1.0	5.1 ± 1.0	5.3 ± 1.0	5.6 ± 1.2	<0.001
TG, mmol/L	1.8 ± 1.3	1.1 ± 0.6	1.5 ± 0.8	2.0 ± 1.1	2.6 ± 1.7	<0.001
eGFR (ml/min per 1.73 m^2^)	88.7 ± 20.1	87.9 ± 20.3	89.4 ± 19.6	89.4 ± 19.9	88.0 ± 20.5	<0.001
Smoking						<0.001
never smokers	7549 (58.6)	1687 (52.7)	1930 (59.9)	2043 (62.9)	1889 (58.7)	
former smokers	1993 (15.5)	515 (16.1)	525 (16.3)	484 (14.9)	469 (14.6)	
current smokers	3351 (26.0)	1001 (31.3)	768 (23.8)	720 (22.2)	862 (26.8)	
Drinking						<0.001
never drinkers	8353 (64.8)	1775 (55.4)	2113 (65.6)	2231 (68.7)	2234 (69.4)	
former drinkers	1628 (12.6)	425 (13.3)	416 (12.9)	392 (12.1)	395 (12.3)	
current drinkers	2912 (22.6)	1003 (31.3)	694 (21.5)	624 (19.2)	591 (18.4)	
CKD, n (%)	612 (4.7)	141 (4.4)	143 (4.4)	149 (4.6)	179 (5.6)	0.094
CHD, n (%)	572 (4.4)	146 (4.6)	142 (4.4)	142 (4.4)	142 (4.4)	0.984
Diabetes, n (%)	2261 (17.5)	380 (11.9)	448 (13.9)	622 (19.2)	811 (25.2)	<0.001
Dyslipidemia, n (%)	8801 (68.3)	1278 (39.9)	1973 (61.2)	2539 (78.2)	3011 (93.5)	<0.001
Atrial fibrillation, n (%)	331 (2.6)	124 (3.9)	70 (2.2)	67 (2.1)	70 (2.2)	<0.001
Antihypertensive drugs, n (%)	8115 (62.9)	1929 (60.2)	2053 (63.7)	2077 (64.0)	2056 (63.9)	0.004
Glucose-lowering drugs, n (%)	591 (4.6)	104 (3.2)	119 (3.7)	168 (5.2)	200 (6.2)	<0.001
Antiplatelet drugs, n (%)	199 (1.5)	54 (1.7%)	44 (1.4%)	48 (1.5%)	53 (1.6%)	0.702

BMI, body mass index; WC, waist circumference; SBP, systolic blood pressure; DBP, diastolic blood pressure; FPG, fasting plasma glucose; Hcy, homocysteine; TG, triglycerides; TC, total cholesterol; LDL-C, low-density lipoprotein cholesterol; HDL-C, high-density lipoprotein cholesterol. eGFR, estimated glomerular filtration rate; CKD, chronic kidney disease; CHD, coronary heart disease.

### Association between LDL-C/HDL-C ratio and stroke

**Figure 2:**
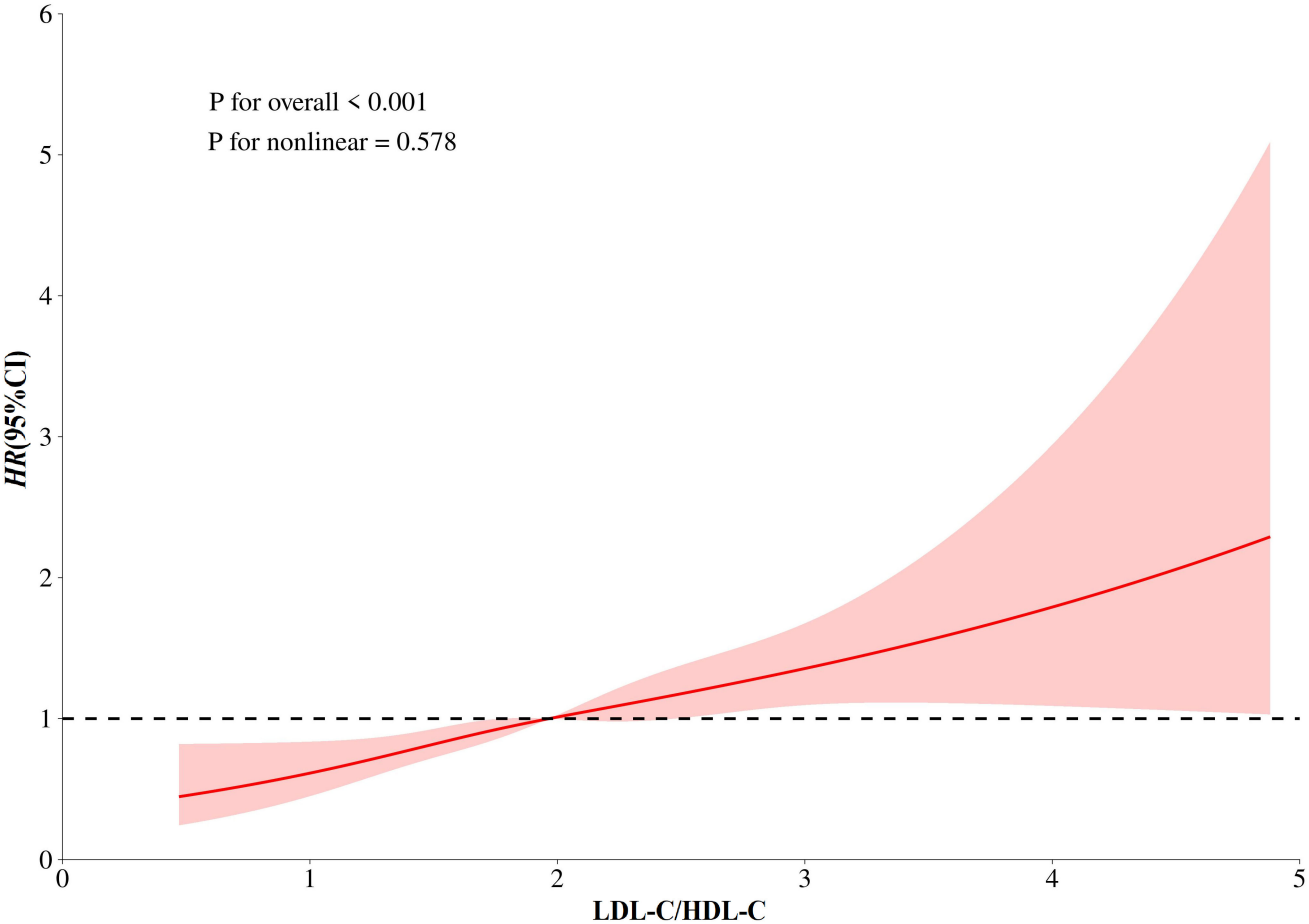
Dose-response relationship between LDL-C/HDL-C Ratio and Stroke. Models were adjusted for sex, age, BMI, WC, SBP, DBP, FPG, TC, TG, eGFR, Hcy, smoking, drinking, diabetes, dyslipidemia, CKD, CHD, AF, antiplatelet drugs, anti-hypertensive drugs, glucose-lowering drugs.

After an average follow-up of 3.9 years, 531 cases of first stroke occurred. In **Figure 2**, the results of the dose-response relationship analysis revealed a positive linear association between LDL-C/HDL-C ratio and first stroke. HRs and 95% CIs of first stroke according to quartiles of LDL-C/HDL-C ratio were summarized in **Table 2**. In the fully adjusted model (Model 3), each 1-unit increment in LDL-C/HDL-C ratio was associated with a 43% increase in the risk of first stroke (HR, 1.43; 95%CI: 1.22, 1.67). When LDL-C/HDL-C ratio were grouped by quartiles, full adjusted HRs of first stroke for patients in quartile 2, quartiles 3 and quartile 4 of LDL-C/HDL-C ratio were 1.32 (95%CI: 1.03,1.70), 1.49 (95%CI: 1.14, 1.96) and 1.94 (95% CI: 1.45, 2.59), compared with those in the quartile 1 (P for trend <0.001). Compared with patients in the combined group of quartile 1 and quartile 2, full adjusted HRs of first stroke for patients in quartiles 3 and quartile 4 of LDL-C/HDL-C ratio were respectively 1.28 (95%CI: 1.02,1.61) and 1.64 (95%CI: 1.29, 2.10).

**Table 2:** Association between LDL-C/HDL-C index and stroke in different models.

LDL-C/HDL-C	N	Events(%)	Stroke, HR (95% CI)		
			Model 1	Model 2	Model 3
Per 1 unit increase	12893	531 (4.1)	1.10 (0.96, 1.25)	1.26 (1.11, 1.43)	1.43 (1.22, 1.67)
Quartiles					
Q1 (0.21-1.54)	3203	130 (4.1)	1.00	1.00	1.00
Q2 (1.55-1.96)	3223	131 (4.1)	1.01 (0.79, 1.29)	1.17 (0.91, 1.49)	1.32 (1.03, 1.70)
Q3 (1.97-2.42)	3247	125 (3.8)	0.98 (0.76, 1.25)	1.22 (0.95, 1.56)	1.49 (1.14, 1.96)
Q4 (2.43-6.51)	3220	145 (4.5)	1.19 (0.94, 1.51)	1.55 (1.22, 1.97)	1.94 (1.45, 2.59)
P for trend			0.198	<0.001	<0.001
Categories					
Q1-Q2 (0.21-2.42)	6426	261 (4.1)	1.00	1.00	1.00
Q3 (1.97-2.42)	3247	127 (3.8)	0.97 (0.78, 1.20)	1.13 (0.91, 1.40)	1.28 (1.02, 1.61)
Q4 (2.43-6.51)	3220	146 (4.5)	1.19 (0.97, 1.45)	1.43 (1.17, 1.76)	1.64 (1.29, 2.10)
P for trend			0.144	<0.001	<0.001

Model 1: adjusted none.

Model 2: adjusted for age, sex.

Model 3: adjusted for age, sex, BMI, WC, SBP, DBP, FPG, TC, TG, eGFR, Hcy, smoking, drinking, diabetes, dyslipidemia, CKD, CHD, AF, antiplatelet drugs, antihypertensive drugs, glucose-lowering drugs.

### Subgroup analysis

We further performed stratified analyses to assess the effect of LDL-C/HDL-C ratio (per 1 unit increment) on first stroke in various subgroups (**Table 3**). The association between LDL-C/HDL-C ratio and first stroke were consistent in the following subgroups: sex (male *vs*. female; *P*-interaction=0.547), age (<65 *vs*. ≥65 y; *P*-interaction=0.556), BMI(<24, ≥24 kg/m^2^; *P*-interaction=0.668), smoking (never smokers *vs*. former smokers *vs*. current smokers; *P*-interaction=0.473), diabetes (no *vs*. yes; *P*-interaction=0.401), and dyslipidemia (no *vs*. yes; *P*-interaction=0.906). However, there was a significant interaction between LDL-C/HDL-C ratio and drinking on first stroke. A stronger positive association between LDL-C/HDL-C ratio and first stroke was found in former drinkers (HR, 1.56; 95%CI: 1.11, 2.19) and current drinkers (HR, 1.76; 95%CI: 1.38, 2.25) compared with never drinkers (HR, 1.28; 95%CI: 1.06, 1.55; *P*-interaction=0.024).

**Table 3:** Subgroup analyses of the effect of LDL-C/HDL-C ratio on stroke.

Subgroup	N	Events, n(%)	HR(95%CI)	*P* for interaction
Sex				0.547
male	5999	308 (5.2)	1.48 (1.24, 1.78)	
female	6894	223 (3.2)	1.37 (1.09, 1.72)	
Age, years				0.556
<65	6431	183 (2.9)	1.36 (1.08, 1.72)	
≥65	6462	348 (5.3)	1.47 (1.23, 1.76)	
BMI,kg/m^2^				0.668
<24	7272	351 (4.8)	1.46 (1.22, 1.75)	
≥24	5621	180 (3.2)	1.38 (1.08, 1.76)	
Smoking				0.473
never smokers	7549	254 (3.4)	1.36 (1.10, 1.69)	
former smokers	1993	86 (4.3)	1.51 (1.10, 2.07)	
current smokers	3351	191 (5.7)	1.50 (1.21, 1.86)	
Drinking				0.024
never drinkers	8535	308 (3.7)	1.28 (1.06, 1.55)	
former drinkers	1628	80 (4.9)	1.56 (1.11, 2.19)	
current drinkers	2912	143 (4.9)	1.76 (1.38, 2.25)	
Diabetes				0.401
no	10632	421 (4.0)	1.48 (1.25, 1.75)	
yes	2261	110 (4.8)	1.30 (0.96, 1.74)	
Dyslipidemia				0.957
no	4092	188 (4.6)	1.47 (1.08, 2.00)	
yes	8801	343 (3.9%)	1.46 (1.23, 1.74)	

Each subgroup analysis adjusted, if not stratified, for sex, age, BMI, WC, SBP, DBP, FPG, TC, TG, eGFR, Hcy, smoking, drinking, diabetes, dyslipidemia, CKD, CHD, AF, antiplatelet drugs, antihypertensive drugs, glucose-lowering drugs. The P-interaction was evaluated against the Bonferroni-corrected α value to minimize the risk of type I error.

## Discussion

In this prospective cohort study of 12,893 hypertensive patients free of prior stroke, we demonstrated that an elevated LDL-C/HDL-C ratio was independently associated with a 43% increased risk of first stroke per 1-unit increment after comprehensive adjustment for cardiovascular risk factors. Notably, this association exhibited a linear dose-response relationship, was significantly potentiated among current drinkers.

This result is consistent with previous findings on lipid ratios and cardiovascular risk, as Zhang et al (29). found in a study of 3469 participants that the risk of stroke in the highest quartile of the LDL-C/HDL-C ratio was 23.45 times higher than in the lowest quartile (OR = 24.45, 95% CI: 17.18-34.79), and the risk increased by the increment of quartile of LDL-C/HDL-C ratio. Liu et al. (30) further revealed the “time-dependent” role of LDL-C/HDL-C ratios in stroke prognosis in a study of 3410 participants, suggesting that acute lipid modulation may improve short-term outcomes, but that long-term high LDL-C/HDL-C ratios remain an important target for intervention in stroke prevention. A large number of evidence supports (11, 12, 31) that the LDL-C/HDL-C ratio has significant predictive value in the assessment of cardiovascular disease risk. In a prospective cohort study of 5099 Chinese rural hypertensive patients, Zheng et al (32) found that the LDL-C/HDL-C ratio was significantly associated with the risk of ischemic stroke, with a predictive efficacy that exceeded that of traditional lipid markers. A prospective cohort study investigating the interaction between dyslipidemia and hypertension in ischemic stroke reported that when stratifying participants into two groups according to LDL-C/HDL-C (≥2 *vs*. <2), and adjusting for age, sex, BMI, smoking, alcohol consumption, and other questionnaire-based covariates, the LDL-C/HDL-C ratio was significantly associated with ischemic stroke risk (HR = 1.414, 95% CI: 1.034–1.933) (33). By contrast, our study provides the first evidence of a continuous, linear association between this ratio and incident stroke in a strictly stroke-naïve cohort. Although our group previously published an analysis of mortality outcomes using the same registry (34), the present investigation focuses specifically on first stroke events with extended follow-up, thereby offering novel pathophysiological insights into primary prevention. Furthermore, based on a large longitudinal study of 384,093 participants from the UK Biobank database, with a median of 11.9 years follow-up, Yuan (35) et al. reported a nonlinear relationship between the reciprocal of LDL-C/HDL-C ratio and stroke: compared with LDL-C/HDL-C=1.67-2.50, LDL-C/HDL-C > 2.5 was correlated with a higher ischemic stroke risk, and LDL-C/HDL-C <1.67 was correlated with a higher hemorrhagic stroke risk after full multivariate adjustment. The conclusion of this study differed from ours, which might be due to the fact that this research was conducted among the British healthy population.

The current academic opinion on the association between alcohol consumption and stroke risk remains controversial, and this association shows significant differences depending on the type of stroke (36). Previous studies have shown (37) that heavy drinking and alcohol abuse are independent risk factors for ischemic stroke, especially in young men. The Meta-analysis system of Patra et al. (38) assessed the dose-effect relationship between alcohol consumption and stroke risk: the risk of ischemic stroke showed a J-shaped curve (small amounts of alcohol may be protective, whereas large amounts increase the risk), whereas the risk of hemorrhagic stroke showed a monotonically increasing trend with increasing alcohol consumption. This finding is partially consistent with the observations of the present study, but no protective effect of alcohol on stroke was found in the present study, and this discrepancy may be related to the higher prevalence of hypertension in the study population. Although the study by Yang (39) et al. reported that small amounts of low-frequency alcohol consumption (≤30 g/day, <5 days/week) were associated with a reduced risk of ischemic stroke at short-term follow-up (≤7 years), this protective effect disappeared at long-term follow-up (>7 years), and the risk of stroke was significantly higher in high-frequency drinkers. This finding suggests that in the long term, alcohol consumption does not reduce stroke risk, regardless of the drinking pattern. Existing studies (40–43) have shown that alcohol consumption may be associated with increased levels of high-density lipoprotein cholesterol and decreased levels of fibrinogen, which may explain the potential association between mild-to-moderate alcohol consumption and a reduced risk of ischemic stroke, but this association does not apply to hemorrhagic stroke. For hemorrhagic stroke (44), alcohol may increase risk by affecting blood pressure. The findings of Kiyohara et al. in the Kuyama-cho study (45) in Japan further support the idea that there is a significant synergistic effect between hypertension and heavy alcohol consumption. Individuals with hypertension combined with heavy alcohol consumption had a significantly increased risk of cerebral hemorrhage compared with nondrinkers; at the same time, the risk of cerebral infarction was twice as high in hypertensive heavy drinkers as in light drinkers. These results show that alcohol consumption may amplify the effects of hypertension on stroke (46). Therefore, the coexistence of alcohol consumption and hypertension warrants special consideration in stroke prevention strategies. Current evidence suggests that hypertensive patients should avoid alcohol consumption to mitigate stroke risk.

Our investigation provides the first evidence of a continuous, linear association between LDL-C/HDL-C ratio and incident stroke in a strictly stroke-naive hypertensive cohort, with comprehensive adjustment for cardiometabolic confounders. This analysis benefits from long-term follow-up in a large cohort, ensuring robust statistical power. Some potential limitations of our study should be noted. First, our study relied on baseline lipid measurements without dynamic monitoring, potentially affecting long-term risk estimation. Second, despite comprehensive adjustment for known confounders, residual confounding from unmeasured variables cannot be entirely ruled out. Finally, Restricted generalizability due to exclusive focus on rural hypertensive populations in Jiangxi Province—extrapolation to urban settings, other regions. Nevertheless, these findings offer clinically actionable guidance for stroke prevention through lipid ratio management in hypertensive patients, warranting validation via future multicenter urban-rural comparative studies.

## Conclusion

Our study demonstrates that an elevated LDL-C/HDL-C ratio—a superior marker of atherogenic/antiatherogenic lipoprotein balance compared to isolated LDL-C or HDL-C measurements—independently associates with increased first stroke risk in hypertensive patients, persisting after adjustment for traditional cardiovascular risk factors and other potential confounders. These findings support the potential clinical utility of the LDL-C/HDL-C ratio as a stroke risk biomarker. Furthermore, our results highlight the importance of incorporating both lipid ratio management and blood pressure control in hypertension treatment strategies. Future research should further validate the predictive value of the LDL-C/HDL-C ratio for stroke prevention and elucidate its underlying biological mechanisms.

## Data Availability

The original contributions presented in the study are included in the article/supplementary material. Further inquiries can be directed to the corresponding authors.
